# Construction of a 3-year risk prediction model for developing diabetes in patients with pre-diabetes

**DOI:** 10.3389/fendo.2024.1410502

**Published:** 2024-06-13

**Authors:** Jianshu Yang, Dan Liu, Qiaoqiao Du, Jing Zhu, Li Lu, Zhengyan Wu, Daiyi Zhang, Xiaodong Ji, Xiang Zheng

**Affiliations:** ^1^ Health Management Center, The First Affiliated Hospital of Soochow University, Suzhou, China; ^2^ Health Management Center, The Second Affiliated Hospital of Soochow University, Suzhou, China

**Keywords:** prediabetes, prediction model, nomogram, HDL-C, physical examination

## Abstract

**Introduction:**

To analyze the influencing factors for progression from newly diagnosed prediabetes (PreDM) to diabetes within 3 years and establish a prediction model to assess the 3-year risk of developing diabetes in patients with PreDM.

**Methods:**

Subjects who were diagnosed with new-onset PreDM at the Physical Examination Center of the First Affiliated Hospital of Soochow University from October 1, 2015 to May 31, 2023 and completed the 3-year follow-up were selected as the study population. Data on gender, age, body mass index (BMI), waist circumference, etc. were collected. After 3 years of follow-up, subjects were divided into a diabetes group and a non-diabetes group. Baseline data between the two groups were compared. A prediction model based on logistic regression was established with nomogram drawn. The calibration was also depicted.

**Results:**

Comparison between diabetes group and non-diabetes group: Differences in 24 indicators including gender, age, history of hypertension, fatty liver, BMI, waist circumference, systolic blood pressure, diastolic blood pressure, fasting blood glucose, HbA1c, etc. were statistically significant between the two groups (P<0.05). Differences in smoking, creatinine and platelet count were not statistically significant between the two groups (P>0.05). Logistic regression analysis showed that ageing, elevated BMI, male gender, high fasting blood glucose, increased LDL-C, fatty liver, liver dysfunction were risk factors for progression from PreDM to diabetes within 3 years (P<0.05), while HDL-C was a protective factor (P<0.05). The derived formula was: In(p/1-p)=0.181×age (40-54 years old)/0.973×age (55-74 years old)/1.868×age (≥75 years old)-0.192×gender (male)+0.151×blood glucose-0.538×BMI (24-28)-0.538×BMI (≥28)-0.109×HDL-C+0.021×LDL-C+0.365×fatty liver (yes)+0.444×liver dysfunction (yes)-10.038. The AUC of the model for predicting progression from PreDM to diabetes within 3 years was 0.787, indicating good predictive ability of the model.

**Conclusions:**

The risk prediction model for developing diabetes within 3 years in patients with PreDM constructed based on 8 influencing factors including age, BMI, gender, fasting blood glucose, LDL-C, HDL-C, fatty liver and liver dysfunction showed good discrimination and calibration.

## Introduction

Diabetes is one of the most common chronic basic diseases at present, and about 90% of them are type 2 diabetes (T2DM) (1). It can cause a variety of metabolic disorders, such as hyperglycemia, hyperlipidemia, insulin resistance (IR), etc., and also lead to Fserious complications, such as diabetes nephropathy (DN), diabetes retinopathy, diabetes peripheral neuropathy, cardiovascular disease (CVD), etc. (2), which is very harmful. The data released by the international diabetes alliance in 2021 shows that about 537 million people aged 20–79 in the world suffer from T2DM (accounting for 10.5% of the total population), and the number is expected to rise to 783 million by 2024, which will bring a heavy burden to the global social and economic development (3). The incidence rate of T2DM in China is about 11.2%, ranking first in the world in terms of the number of patients. Therefore, the prevention and treatment of T2DM is extremely urgent.

Prediabetes mellitus (PreDM) is an intermediate stage of dysglycemia preceding diabetes mellitus, and it is associated with increased risks of cardiovascular disease, microvascular complications, tumors, dementia, depression, etc. (4). About 5–10% of PreDM cases progress to diabetes annually, and without intervention, over 70% of PreDM would finally advance to diabetes (5, 6). In the study in Da Qing, China, the cumulative incidence rate of diabetes reached 95.9% in PreDM patients after a 30-year follow-up; while for PreDM patients with impaired glucose tolerance receiving lifestyle intervention for 6 years, their 30-year cumulative risk of developing diabetes decreased by 39% (6). Therefore, early screening for PreDM and intervention on high-risk populations can delay the progression of diabetes and prevent the incidence of diabetes.

With the development of economy, in nowadays, researchers have paid more attention to the physical examinations (7, 8). Finding valuable information related to diabetes from physical examination data and finding out the changing pattern of diabetes at all stages is of great importance to the prevention and treatment of diabetes (9). For example, during the physical examination procedure, gender, age, body mass index (BMI), waist circumference, past medical history, smoking history, systolic blood pressure (SBP), diastolic blood pressure (DBP), pulse rate, white blood cells (WBC), red blood cells (RBC), hemoglobin (Hb), platelet count (PLT), total cholesterol (TC), triglycerides (TG), high density lipoprotein cholesterol (HDL-C), low density lipoprotein cholesterol (LDL-C), blood glucose, glycated hemoglobin (HbA1c), creatinine (Cr), estimated glomerular filtration rate (eGFR), uric acid, alanine aminotransferase (ALT), aspartate aminotransferase (AST), γ-glutamyltransferase (γ-GT), level of urine protein and whether the patient has fatty liver were always collected for the diagnosis of diabetes. Moreover, risk factors associated with diabetes have been discussed in many previous studies. For example, in the past, the onset age of diabetes was mainly middle-aged and elderly people over 50 years old, but now it is mainly concentrated in young adults aged 20 to 40 years old, which is closely related to the current unhealthy lifestyle of young people; moreover, type 2 diabetes is highly correlated with obesity, and an obese family (BMI ≥ 30) has a rapidly increasing risk of diabetes since the age of 35; further, genetic susceptibility is a characteristic of diabetes, and is also affected by other factors, such as living habits and family environment (10). The incidence rate of people without diabetes family history is lower than that of people with diabetes family history, so we should focus on this part of people with diabetes family history in the early stage who have diabetes family history (11); One of the important factors of diabetes is the abnormal increase of triglycerides. The basic reasons are: The triglycerides and free fatty acids increase at the same time, and the islets of langerhans β, the main component of cellular regulation is free fatty acids, and their elevation causes abnormal insulin secretion, leading to abnormal elevation of blood sugar Elevated triglycerides induce an exacerbation of insulin resistance and exacerbate dyslipidemia, leading to a vicious cycle. At present, diabetes is a metabolic disease with a very high incidence rate and a very low treatment compliance rate. Poor control can lead to the occurrence of various acute and chronic complications Therefore, it important for an early detection of diabetes for improving the quality of life of the patients.

In recent years, researchers have developed models for the prediction of diabetes. However, studies on developing models for the prediction of diabetes are still required for the early diagnosis and management of diabetes. In current work, we intend to construct a prediction model for evaluating the progression from PreDM to diabetes mellitus within 3 years. By screening PreDM patients through health examinations and following them up for 3 years, high-risk factors for diabetes occurrence will be analyzed to identify high-risk patients and formulate targeted strategies, so as to reduce the risk of diabetes.

## Materials and methods

### Study subjects

**Figure 1:**
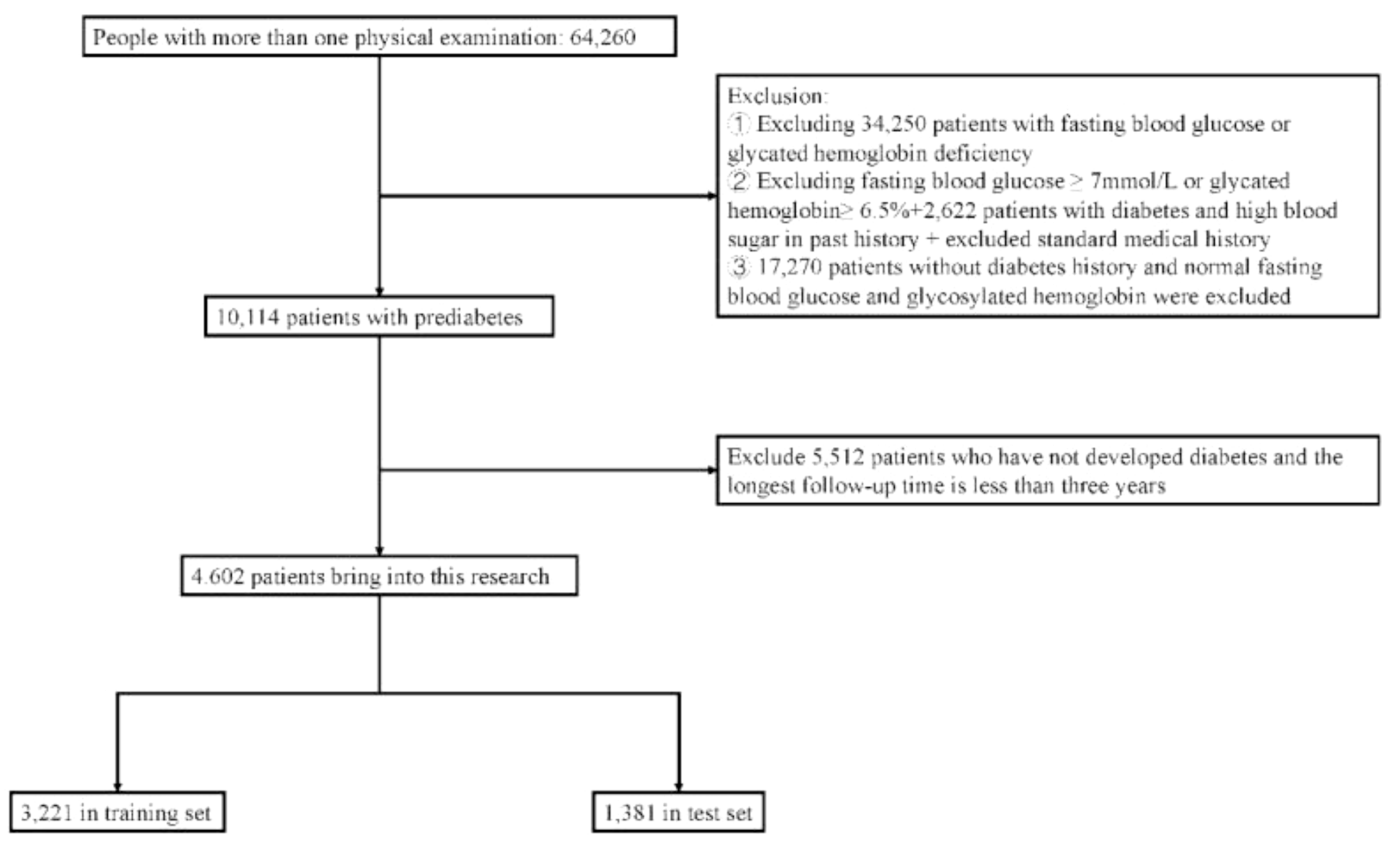
Research object selection process.

Data from the Physical Examination Center of the First Affiliated Hospital of Soochow University from October 1, 2015 to May 31, 2023 were selected. Newly diagnosed PreDM cases were screened and followed up for 3 years. A total of 4,602 PreDM patients were finally enrolled. Inclusion criteria: 1) Age ≥20 years old; 2) Fasting blood glucose and HbA1c data must be available in the physical examination; 3) 6.1≤fasting blood glucose<7.0 mmol/L and/or 5.7%≤HbA1c<6.5%; 4) No previous history of diabetes or hyperglycemia; 5) Not lost to follow-up, and must have physical examination data in the 4th year, or developed diabetes within 3 years. Exclusion criteria: 1) Use of hypoglycemic agents or steroid hormones within 1 year; 2) Severe liver and kidney dysfunction; 3) Severe anemia, hemoglobin disease, pregnancy, AIDS, malignant tumors; 4) Recurrent acute pancreatitis, history of acute pancreatitis in the last 3 months, history of surgery in the last 3 months. A completely random sampling method was adopted, with 70% of the included population as the training set and 30% as the test set (**Figure 1**).

### Data collection

The clinical data included: gender, age, body mass index (BMI), waist circumference, past history (history of hypertension, diabetes, high blood glucose), smoking history, systolic blood pressure (SBP), diastolic blood pressure (DBP), pulse rate, white blood cells (WBC), red blood cells (RBC), hemoglobin (Hb), platelet count (PLT), total cholesterol (TC), triglycerides (TG), high density lipoprotein cholesterol (HDL-C), low density lipoprotein cholesterol (LDL-C), blood glucose, glycated hemoglobin (HbA1c), creatinine (Cr), estimated glomerular filtration rate (eGFR), uric acid, alanine aminotransferase (ALT), aspartate aminotransferase (AST), γ-glutamyltransferase (γ-GT), urine protein (0 for negative, 1 for positive), fatty liver (0 for no, 1 for yes). The collected data were from the first-year physical examination results of the new-onset PreDM population. For the missing value of variables, imputation was done using regression method, that each variable was estimated 100 times and the mean value was using in the analysis.

### Outcomes

The endpoint of this study was the onset of diabetes in the subjects. Diabetes was defined as FPG≥7.0 mmol/L or 2hPG≥11.1 mmol/L. According to WHO’s recommendation in 2011, HbA1c≥6.5% was supplemented as a diagnostic criterion for diabetes.

### Statistical methods

Statistical analysis was performed using SAS 9.4 software and R software version 4.2.3. Normally distributed measurement data were expressed as mean ± standard deviation, non-normally distributed data were expressed as median (lower quartile, upper quartile), and qualitative data were expressed as frequency (percentage). Differences between groups for numerical variables were compared using the Kruskal-Wallis nonparametric test and t-test. Differences between groups for categorical variables were compared using Chi-square test. A two-sided test of P<0.05 indicated statistically significant difference.

### Model development and validation

Logistic regression was used for predictor variable selection on the training dataset, and variables that could serve as predictive factors were selected using the stepwise method (p value threshold of 0.2 for adding variables and 0.1 for removing variables). Considering the practical application value of the predictive model, we combined the results of the model selection and the clinical significance to determine the variables that ultimately served as predictors. Based on the predictors, we established a multivariate logistic regression model and reported the model parameters. Equations was utilized to construct the prediction model. The area under the curve (AUC) and 95% confidence interval was reported to evaluate discrimination. The slope (intercept) of the decile calibration curve was used to report calibration, calculated by regressing the observed outcome on the predicted probabilities. A slope closer to 1 and intercept closer to 0 represented better calibration power.

The parameters of the predictive model described above were applied to the validation dataset to validate the model. Similarly, the discrimination (AUC and 95%CI) and calibration (slope and intercept) ability of the model in the validation group are reported. Additionally, an internal validation based on the training group data after 1,000 resampling was performed, and the adjusted AUC was reported. To assess the heterogeneity within different subpopulations, we performed sensitive analysis among selected subgroups, including age (<50 and >=50 years), sex, BMI (<28 and >=28), whether having hypertension history, smoking, or whether having fatty liver.

### Model application

A nomogram was depicted to present the results visually, and the calibration of the nomogram was calculated with a calibration curve plotted. Model clinical decision curve and clinical impact curve were also plotted to provided further information.

## Results

### Comparison between diabetic and non-diabetic PreDM groups

From 2017 to 2023, a total of 4,602 PreDM samples were enrolled in the study, among which 760 participants (16.51%) developed diabetes within 3 years, and 3,842 participants (83.49%) did not. Results for comparison between the diabetic and non-diabetic PreDM groups showed that 24 indicators, including gender, age, history of hypertension, fatty liver, BMI, etc. were statistically significant between the two groups (all P<0.05). On the other hand, differences in smoking, creatinine and platelet count were not statistically significant (**Table 1**).

**Table 1:** Comparison of the baseline data of PreDM population with or without diabetes.

	PreDM group	Diabetes in 3 years	No diabetes in 3 years	P value
	N=4602	N=760	N=3842	
Gender[n(%)]				
Male	3062(66.54)	573(75.39)	2489(64.78)	<0.01
Female	1540(33.46)	187(24.61)	1353(35.22)	.
Months between diabetes	123	23(12, 28)	37(37, 37)	
Baseline age (years)	51.65 ± 12.54	55.44 ± 13.36	50.9 ± 12.23	<0.01
18–39	772(16.78)	85(11.18)	687(17.88)	<0.01
40–54	2194(47.67)	298(39.21)	1896(49.35)	.
55–74	1407(30.57)	309(40.66)	1098(28.58)	.
≥75	229(4.98)	68(8.95)	161(4.19)	.
History of Hypertension	1068(23.94)	257(35.11)	811(21.74)	<0.01
Smoke	1925(41.83)	322(42.37)	1603(41.72)	0.74
Fatty Liver	1842(40.03)	445(58.55)	1397(36.36)	<0.01
BMI(kg/m2)	24.96 ± 3.21	26.14 ± 3.26	24.72 ± 3.15	<0.01
BMI<24	1729(37.57)	188(24.74)	1541(40.11)	<0.01
24≤BMI<28	2155(46.83)	367(48.29)	1788(46.54)	.
BMI≥28	718(15.6)	205(26.97)	513(13.35)	.
waistline (cm)	87.16 ± 9.69	91.28 ± 9.29	86.34 ± 9.56	<0.01
≥90(Male)/≥85(Female)	2131(46.31)	473(62.24)	1658(43.15)	<0.01
Systolic Pressure (mmHg)	128.28 ± 18.08	135.02 ± 18.98	126.94 ± 17.6	<0.01
SBP<120	1562(33.94)	171(22.5)	1391(36.21)	<0.01
120≤SBP<140	1924(41.81)	311(40.92)	1613(41.98)	.
140≤SBP<160	867(18.84)	200(26.32)	667(17.36)	.
SBP≥160	249(5.41)	78(10.26)	171(4.45)	.
Diastolic pressure (mmHg)	77.65 ± 11.23	80.89 ± 11.71	77.01 ± 11.03	<0.01
DBP<60	219(4.76)	22(2.89)	197(5.13)	<0.01
60≤DBP<90	3735(81.16)	568(74.74)	3167(82.43)	.
90≤DBP<100	498(10.82)	128(16.84)	370(9.63)	.
DBP≥100	150(3.26)	42(5.53)	108(2.81)	.
FBS (mmol/L)	5.47 ± 0.56	5.88 ± 0.6	5.39 ± 0.52	<0.01
Glycosylated Hemoglobin (%)	5.89 ± 0.21	6.12 ± 0.22	5.85 ± 0.18	<0.01
Total Cholesterol (mmol/L)	5.01 ± 0.91	5.08 ± 0.97	5 ± 0.89	0.04
High Density Cholesterol (mmol/L)	1.36 ± 0.32	1.25 ± 0.3	1.38 ± 0.32	<0.01
Non High-Density Lipoprotein (mmol/L)	3.66 ± 0.88	3.83 ± 0.91	3.62 ± 0.87	<0.01
Low Density Cholesterol (mmol/L)	3 ± 0.78	3.09 ± 0.85	2.99 ± 0.77	<0.01
Triglyceride (mmol/L)	1.77 ± 1.21	2.06 ± 1.29	1.71 ± 1.19	<0.01
TG/HDL-C	1.48 ± 1.4	1.83 ± 1.47	1.41 ± 1.37	<0.01
Creatinine (umol/L)	69.65 ± 15.69	70.63 ± 15.07	69.46 ± 15.8	0.06
Glomerular Filtration Rate EGFR	99.98 ± 13.97	97.62 ± 14.62	100.44 ± 13.79	<0.01
Uric Acid (mg/dl)	61.24 ± 14.73	65.31 ± 14.41	60.44 ± 14.66	<0.01
Uric Acid Q1	948(20.6)	105(13.82)	843(21.94)	<0.01
Uric Acid Q2	1259(27.36)	193(25.39)	1066(27.75)	.
Uric Acid Q3	1219(26.49)	207(27.24)	1012(26.34)	.
Uric Acid Q4	1176(25.55)	255(33.55)	921(23.97)	.
WBC(10^9^/L)	6.31 ± 1.59	6.58 ± 1.65	6.25 ± 1.57	<0.01
RBC(10^12^/L)	4.84 ± 0.47	4.88 ± 0.46	4.83 ± 0.48	<0.01
Hemoglobin (g/L)	145.45 ± 15.5	148.57 ± 15.08	144.83 ± 15.5	<0.01
Platelet Count (10^9^/L)	229.28 ± 56.2	228.63 ± 58.52	229.41 ± 55.74	0.73
ALT	22.4(16.7,32.8)	25.3(18.15,38.25)	21.9(16.3,31.8)	<0.01
AST	21.3(18.2,25.6)	22.4(19,27.95)	21(18,25.1)	<0.01
γ-GTP	26.1(17.4, 42)	32.85(22, 51.7)	24.9(16.7,40.5)	<0.01

### Prediction model for 3-year risk of developing diabetes in PreDM patients

Among the variables with P<0.05, fasting blood glucose, HDL-C and LDL-C were selected as candidates. Moreover, age (20–39 years old, 40–54 years old, 55–74 years old, ≥75 years old), gender (male, female), BMI (BMI<24, 24≤BMI<28, BMI≥28), fatty liver (yes, no), liver dysfunction (ALT >50 + AST >40) were selected as the candidate categorical variables.

Results of logistic regression analysis showed that increased age, male gender, elevated BMI, high blood glucose, increased LDL-C, fatty liver, liver dysfunction were risk factors for progression from prediabetes to diabetes within 3 years (P<0.05), while HDL-C was a protective factor (P<0.05). The derived formula was: In(p/1-p)=0.181×age (40–54 years old)/0.973×age (55–74 years old)/1.868×age (≥75 years old)-0.192×gender (male)+0.151×blood glucose-0.538×BMI (24–28)-0.538×BMI (≥28)-0.109×HDL-C+0.021×LDL-C+0.365×fatty liver (yes)+0.444×liver dysfunction (yes)-10.038. The results were shown in **Table 2**.

**Table 2:** Logistic regression analysis of risk factors affecting the progression of PreDM patients to diabetes in three years.

Element	Regression Coefficient	Standard Error	Wald *χ* ^2^ Value	P Value	OR Value	95% CI
Age						
40–54	0.181	0.175	1.070	0.301	1.200	0.85,1.69
55–74	0.973	0.182	28.757	0.000	2.650	1.85,3.78
≥75	1.868	0.259	51.972	0.000	6.470	3.9,10.75
Gender(Male)	-0.192	0.128	2.237	0.135	0.830	0.64,1.06
Blood Glucose(per 0.1mmol/L)	0.151	0.010	225.325	0.000	1.160	1.14,1.19
BMI						
24–28	-0.058	0.137	0.178	0.673	0.940	0.72,1.23
≥28	0.538	0.170	10.005	0.002	1.710	1.23,2.39
HDL-C(PER 0.1mmol/L)	-0.109	0.020	28.806	0.000	0.900	0.86,0.93
LDL-C(PER 0.1mmol/L)	0.021	0.007	9.578	0.002	1.020	1.01,1.03
Fatty Liver	0.365	0.123	8.744	0.003	1.440	1.13,1.83
ALT>50/AST>40	0.444	0.184	5.816	0.016	1.560	1.09,2.24

**Figure 2:**
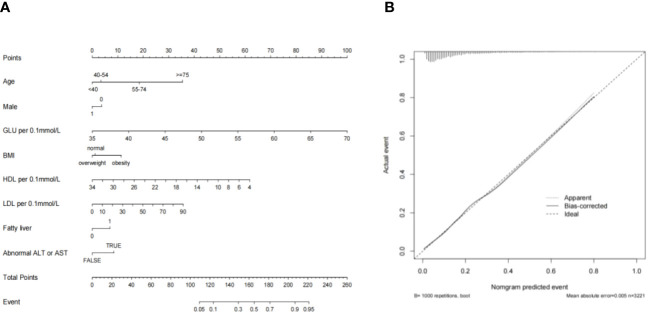
**(A)** Predictive Model Column Chart; **(B)** Column chart calibration curve.

Based on the logistic regression results, a nomogram predicting 3-year risk of progression from prediabetes to diabetes was constructed using R software for visualization (**Figure 2A**). In the nomogram, each specific situation of the risk factors corresponds to a certain score. The total score is calculated by adding up the scores of the 6 indicators. Then a vertical line has been drawn downward at the location of the total score, and the corresponding value of the intersection point between the vertical line and the “probability of diabetes occurrence” coordinate is the 3-year risk of progression from PreDM to diabetes. The calibration curve of the nomogram was also been plotted (**Figure 2B**).

**Figure 3:**
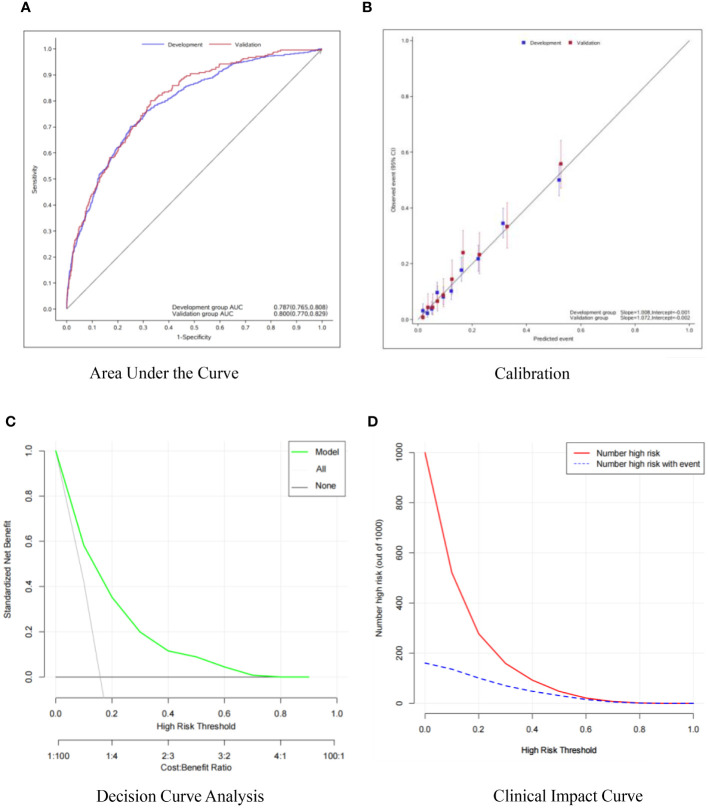
**(A)** AUC plot; **(B)** calibration plot; **(C)** clinical decision curve; **(D)** impact curve.

The AUC of the training model ROC curve = 0.787 (95%CI: [0.765, 0.808]), AUC of internal validation on testing set = 0.800 (95%CI: [0.770, 0.829]) (**Figure 3A**), indicating good predictive ability of the model. The calibration slope = 1.008 and intercept = -0.001 suggested good calibration (**Figure 3B**). The model accuracy plots were verified by clinical decision curve (**Figure 3C**) and clinical impact curve (**Figure 3D**). Results from sensitive analysis showed quite stable AUC and calibration slope among different subgroups (**Table 3**).

**Table 3:** Model performance among subgroups.

Groups	Subgroups	Number of patients	Number of event(%)	Rate(95% CI) of event	AUC(95% CI)	Calibration slope (Intercept)
**Training dataset**	Age<50,years	1421	168(11.82)	11.82(10.19–13.62)	0.823(0.788–0.858)	1.11(-0.022)
	Age>=50,years	1800	350(19.44)	19.44(17.64–21.35)	0.752(0.723–0.780)	0.95(0.016)
	Male	2123	388(18.28)	18.28(16.65–19.99)	0.792(0.767–0.817)	1.07(-0.012)
	Female	1098	130(11.84)	11.84(9.99–13.90)	0.761(0.717–0.804)	0.83(0.02)
	BMI<28	2731	379(13.88)	13.88(12.60–15.23)	0.787(0.762–0.812)	1.06(-0.008)
	BMI >=28	490	139(28.37)	28.37(24.41–32.58)	0.712(0.662–0.763)	0.87(0.038)
	Hypertension	743	172(23.15)	23.15(20.16–26.35)	0.729(0.686–0.771)	0.88(0.028)
	Not Hypertension	2379	326(13.70)	13.70(12.35–15.15)	0.791(0.764–0.818)	1.04(-0.008)
	Smoker	1339	228(17.03)	17.03(15.05–19.15)	0.796(0.765–0.828)	1.05(-0.013)
	Non Smoker	1882	290(15.41)	15.41(13.81–17.12)	0.780(0.751–0.809)	0.98(0.006)
	Fatty liver	1275	298(23.37)	23.37(21.07–25.79)	0.757(0.726–0.788)	1(0)
	Not fatty liver	1946	220(11.31)	11.31(9.93–12.80)	0.775(0.742–0.809)	1.02(-0.002)
**Test dataset**	Age<50,years	609	89(14.61)	14.61(11.90–17.67)	0.832(0.791–0.873)	1.15(-0.001)
	Age>=50,years	772	153(19.82)	19.82(17.06–22.81)	0.777(0.735–0.818)	1.05(-0.006)
	Male	939	185(19.70)	19.70(17.20–22.39)	0.794(0.760–0.829)	1.11(-0.007)
	Female	442	57(12.90)	12.90(9.92–16.38)	0.799(0.740–0.857)	0.94(0.011)
	BMI<28	1153	176(15.26)	15.26(13.24–17.47)	0.797(0.762–0.832)	1.17(-0.011)
	BMI >=28	228	66(28.95)	28.95(23.15–35.30)	0.744(0.679–0.809)	0.91(0.027)
	Hypertension	325	85(26.15)	26.15(21.46–31.29)	0.741(0.685–0.799)	0.91(0.054)
	Not Hypertension	1015	149(14.68)	14.68(12.56–17.01)	0.807(0.770–0.845)	1.17(-0.019)
	Smoker	586	94(16.04)	16.04(13.16–19.27)	0.790(0.741–0.838)	1(-0.007)
	Non Smoker	795	148(18.62)	18.62(15.97–21.50)	0.807(0.771–0.844)	1.13(0.002)
	Fatty liver	567	147(25.93)	25.93(22.36–29.74)	0.727(0.680–0.774)	0.94(0.041)
	Not fatty liver	814	95(11.67)	11.67(9.55–14.08)	0.823(0.779–0.868)	1.19(-0.023)

## Discussion

Diabetes has become one of the leading causes of human death in recent decades (12). The incidence of diabetes increased every year due to eating habits, sedentary lifestyle, and prevalence of unhealthful foods (13, 14). Diabetes prediction model can contribute to the decision-making process in clinical management (15, 16). Knowing the potential risk factors and identifying individuals at high risk in the early stages may facilitate the process for the prevention of diabetes. A host of prediction models for diabetes have been developed, out of which the logistic regression (17) and a machine learning algorithm-based classification tree (18) are among the most popular methods. For example, Habibi et al. suggest that a simple machine learning algorithm, a classification tree, could be used to screen diabetes without using a laboratory (19). However, the validity of these models for different locations, populations with different diets, lifestyle, races, and genetic makeup is still unknown. Additionally, the performance of the models varied in different circumstances.

Moreover, the International Diabetes Federation estimates that the number of adults with impaired glucose tolerance will reach 730 million by 2045, accounting for 11.2% of the world’s adult population (20). The TULIP study in Germany classified PreDM patients into high-risk and low-risk phenotypes (21). Intensified management of the high-risk populations can improve glycemic and cardiac metabolic outcomes, while routine intervention for low-risk populations can avoid unnecessary overtreatment (22). Therefore, individualized and risk-based management of PreDM patients with high coverage is beneficial for slowing of the progression of diabetes and the reduction of diabetes morbidity. In this study, new-onset PreDM cases were screened out in the physical examination population. We successfully established a prediction model and a nomogram predicting 3-year risk of progression from prediabetes to diabetes was depicted to assess diabetes risk. The results suggested that intervention and management of high-risk populations is of positive clinical significance in delaying diabetes progression and reducing diabetes incidence.

Models for the prediction of diabetes have been developed in previous studies. For example, Zou et al. used principal component analysis (PCA) and minimum redundant maximum (mRMR) correlation to screen risk factors, and utilized decision tree (DT), random forest (RF) and neural network (NN) to predict diabetes (23). By using mutual information (MI) and Gini impurity (GI) to screen diabetes-related risk factors in physical examination data, Yang et al. established a cascade diabetes risk prediction system (24). Moreover, the invasive risk assessment model HCL predicted diabetes by using invasive characteristics and referring to Harvard Cancer Risk Index (25). Further, Li et al. established a prediction model for type 2 diabetes for Han Chinese population. The result suggested that genetic risk score is a crucial element to predicting the risk of type 2 diabetes. In conclusion, different prediction models for diabetes have been established and the early diagnosis of diabetes could be achieved; Hu et al. established a nomogram model for the prediction of 5-year risk of prediabetes in Chinese adults, and they suggested that this model could be applied for prediabetes prediction and assessing the risk of prediabetes (26); Cai et al. established a model for the incidence of type 2 diabetes in non-obese patients in 5 years, the authors claimed that the mode is helpful for reducing the risk of T2D in non-obese adults (27); finally, Cai et al. developed a model for predicting the 5-year risk of T2D in hypertension patients, and they found the model could reduce the incidence of T2D in patients with hypertension (28). However, in current clinical field, there are limited studies focused on the short-term progress of type 2 diabetes within three years, and in this study, we first completed a 3-year follow-up, which is a supplement to the T2D related field.

Our results showed that age, gender, increased BMI, high blood glucose level, elevated level of low-density cholesterol, fatty liver, and abnormal liver function were the risk factors for patients with PreDM to progress to diabetes within three years. Many previous studies showed the incident rate of T2DM increase with age. It has been reported that about 4% diabetic patients aged less than 44 years, while the percentage of reached 17.0% for people between 45–64 years, and 25.2% for people ≥65 years (29). Moreover, Peng et al. reported that 16.9% patients had T2DM and a follow-up survey on the same group suggested that the number increased to 23.7% (30). This may due to the decreased sensitivity to insulin with the increase of age. Moreover, the distribution of T2DM was 221.0 million for males and 203.9 million for females, suggesting that T2DM is related to gender (31). Furthermore, 50% T2DM patients are obese (BMI > 30 kg/m2), while 90% diabetic patients are overweight (BMI > 25 kg/m2), suggesting that BMI is a risk factor for proceeding to diabetes. Moreover, high blood glucose level is an early indicator of pre-diabetes to diabetes, when the patient is diagnosed with impaired glucose tolerance (IGT) or impaired fasting glucose (IFG) (32). Next, in PreDM patients, dyslipidemia has been caused by increased LDL-C level and decreased HDL-C levels (33, 34). Aberrantly increased LDL-C as well as decreased HDL-C may lead to dysfunction of the islet β cells, and accelerated the procedure from PreDM to type 2 DM (35). Results of previous studies showed either LDL-C or HDL-C were correlated with the risk of abnormal glucose metabolism. Finally, epidemiological studies showed that there is a clear relationship between fatty liver and the incidence of type 2 diabetes, increased fatty liver index may increase the odds for the incident of PreDM prediabetes. On the other hand, the abnormality liver function is strongly associated with obesity and insulin resistance, as a result, abnormality in liver function has become an independent risk factor for incident T2DM (36–40). To sum up, the result of current study confirmed the risk factors for patients with PreDM to progress to diabetes, however, the results still need to be confirmed with in depth study.

This study still has some limitations: (1) Fewer females with preDM were enrolled, and there was an imbalance in the male-to-female ratio; (2) HbA1c better reflects glucose tolerance, but considering the inadequate standardization of HbA1c testing among different hospitals, it was not used in the prediction model; (3) No external validation was performed. Multi-center studies will be carried out later to further improve the prediction model. At present, only the incidence of diabetes has been observed during the past three years, and in future studies, we will first expand the sample size, and second. there will be more index for observations, such as the complications of diabetes, and the follow-up of the patients will continue to be observed.

In summary, in PreDM patients, good control of body weight, blood lipids, reversal of fatty liver, and maintenance of liver function in the normal range, especially for males over 55 years old, are effective management measures to delay progression from PreDM to diabetes. Timely individualized interventions should be adopted for high-risk PreDM populations to reduce the risk of developing diabetes.

## Data availability statement

The original contributions presented in the study are included in the article/supplementary material. Further inquiries can be directed to the corresponding authors.

## Author contributions

JY: Conceptualization, Investigation, Writing – original draft, Writing – review & editing. DL: Investigation, Writing – original draft, Writing – review & editing. JD: Investigation, Writing – original draft, Writing – review & editing. JZ: Data curation, Investigation, Writing – review & editing. LL: Formal analysis, Methodology, Project administration, Writing – original draft. ZW: Project administration, Software, Supervision, Writing – review & editing. DZ: Data curation, Methodology, Writing – review & editing. XJ: Methodology, Writing – review & editing. XZ: Formal analysis, Supervision, Writing – review & editing.

## References

[1] Shapiro SB Yin H Yu OHY Azoulay L. Dipeptidyl peptidase-4 inhibitors and the risk of gallbladder and bile duct disease among patients with type 2 diabetes: A population-based cohort study. Drug Saf. (2024). doi: 10.1007/s40264-024-01434-4 38720114

[2] Chang CJ Fan YH Chiu YC Cheng WM. Cold hypersensitivity in the hands and feet is associated with erectile dysfunction in young Taiwanese men. Sci Rep. (2024) 14:10577. doi: 10.1038/s41598-024-60260-x 38719920 PMC11078973

[3] Peregrina HN Bayog MLG Pagdilao A Bender MS Doan T Yoo GJ. Older chinese and filipino american immigrants with type 2 diabetes and their adult child: A qualitative dyadic exploration of family support. J Cross-Cult Gerontol.. (2024) 39(2):151–72. doi: 10.1007/s10823-024-09505-w PMC11093813 38720112

[4] De Sanctis V Soliman AT Daar S Tzoulis P Kattamis C. Can we predict incipient diabetes mellitus in patients with transfusion dependent β-thalassemia (β-TDT) referred with a history of prediabetes? Mediterr J Hematol Infect Dis. (2024) 16:e2024005. doi: 10.4084/mjhid.2024.005 38223478 PMC10786125

[5] Abelleira R Zamarrón C Riveiro V Casal A Toubes ME Rábade C. Relationship between obstructive sleep apnea and type 2 diabetes mellitus. Medicina clinica. (2024) 162(8):363–9. doi: 10.1016/j.medcli.2023.11.014 38220552

[6] Gong Q Zhang P Wang J Ma J An Y Chen Y. Morbidity and mortality after lifestyle intervention for people with impaired glucose tolerance: 30-year results of the Da Qing Diabetes Prevention Outcome Study. Lancet Diabetes Endocrinol. (2019) 7:452–61. doi: 10.1016/s2213-8587(19)30093-2 PMC8172050 31036503

[7] Stocks CO Carson RA. Newborn and infant vision screening in primary care: A clinical review. J specialists Pediatr nursing: JSPN. (2024) 29:e12421. doi: 10.1111/jspn.12421 38284218

[8] Chen Y Yan D You N Gu B Wang Q Zhang J. Effect of Helicobacter pylori infection on body fat percentage in middle-aged and elderly populations. Prev Med Rep. (2024) 38:102601. doi: 10.1016/j.pmedr.2024.102601 38283954 PMC10821582

[9] Kim YG Min K Jeong JH Roh SY Han KD Shim J. Temporal elevation of blood pressure is associated with increased risk of sudden cardiac arrest. Sci Rep. (2024) 14:2289. doi: 10.1038/s41598-024-52859-x 38280904 PMC10821940

[10] Di Iorgi N Napoli F Allegri AE Olivieri I Bertelli E Gallizia A. Diabetes insipidus–diagnosis and management. Hormone Res Pediatr. (2012) 77:69–84. doi: 10.1159/000336333 22433947

[11] Refardt J Winzeler B Christ-Crain M. Copeptin and its role in the diagnosis of diabetes insipidus and the syndrome of inappropriate antidiuresis. Clin Endocrinol. (2019) 91:22–32. doi: 10.1111/cen.13991 PMC6850413 31004513

[12] Refardt J Winzeler B Christ-Crain M. Diabetes insipidus: an update. Endocrinol Metab Clinics North America. (2020) 49:517–31. doi: 10.1016/j.ecl.2020.05.012 32741486

[13] Vaz de Castro PAS Bitencourt L de Oliveira Campos JL Fischer BL Soares de Brito SBC Soares BS. Nephrogenic diabetes insipidus: a comprehensive overview. J Pediatr Endocrinol metabolism: JPEM. (2022) 35:421–34. doi: 10.1515/jpem-2021-0566 35146976

[14] Almalki MH Ahmad MM Brema I Almehthel M AlDahmani KM Mahzari M. Management of diabetes insipidus following surgery for pituitary and suprasellar tumors. Sultan Qaboos Univ Med J. (2021) 21:354–64. doi: 10.18295/squmj.4.2021.010 PMC8407907 34522399

[15] Bu F Deng XH Zhan NN Cheng H Wang ZL Tang L. Development and validation of a risk prediction model for frailty in patients with diabetes. BMC geriatrics. (2023) 23:172. doi: 10.1186/s12877-023-03823-3 36973658 PMC10045211

[16] Zou Y Zhao L Zhang J Wang Y Wu Y Ren H. Development and internal validation of machine learning algorithms for end-stage renal disease risk prediction model of people with type 2 diabetes mellitus and diabetic kidney disease. Renal failure. (2022) 44:562–70. doi: 10.1080/0886022x.2022.2056053 PMC8986220 35373711

[17] Joshi RD Dhakal CK. Predicting type 2 diabetes using logistic regression and machine learning approaches. Int J Environ Res Public Health. (2021) 18(14):7346. doi: 10.3390/ijerph18147346 34299797 PMC8306487

[18] Dinh A Miertschin S Young A Mohanty SD. A data-driven approach to predicting diabetes and cardiovascular disease with machine learning. BMC Med Inf decision making. (2019) 19:211. doi: 10.1186/s12911-019-0918-5 PMC6836338 31694707

[19] Habibi N Mousa A Tay CT Khomami MB Patten RK Andraweera PH. Maternal metabolic factors and the association with gestational diabetes: A systematic review and meta-analysis. Diabetes/metabolism Res Rev. (2022) 38:e3532. doi: 10.1002/dmrr.3532 PMC9540632 35421281

[20] Saeedi P Petersohn I Salpea P Malanda B Karuranga S Unwin N. Global and regional diabetes prevalence estimates for 2019 and projections for 2030 and 2045: Results from the International Diabetes Federation Diabetes Atlas, 9(th) edition. Diabetes Res Clin Pract. (2019) 157:107843. doi: 10.1016/j.diabres.2019.107843 31518657

[21] Muralidhara S Lucieri A Dengel A Ahmed S. Holistic multi-class classification & grading of diabetic foot ulcerations from plantar thermal images using deep learning. Health Inf Sci Syst. (2022) 10:21. doi: 10.1007/s13755-022-00194-8 36039095 PMC9418397

[22] Yang T Liu Y Li L Zheng Y Wang Y Su J. Correlation between the triglyceride-to-high-density lipoprotein cholesterol ratio and other unconventional lipid parameters with the risk of prediabetes and Type 2 diabetes in patients with coronary heart disease: a RCSCD-TCM study in China. Cardiovasc Diabetol. (2022) 21:93. doi: 10.1186/s12933-022-01531-7 35659300 PMC9166647

[23] Zou Q Qu K Luo Y Yin D Ju Y Tang H. Predicting diabetes mellitus with machine learning techniques. Front Genet. (2018) 9:515. doi: 10.3389/fgene.2018.00515 30459809 PMC6232260

[24] Yang P Li X Xu C Eckert RL Reece EA Zielke HR. Maternal hyperglycemia activates an ASK1-FoxO3a-caspase 8 pathway that leads to embryonic neural tube defects. Sci Signaling. (2013) 6:ra74. doi: 10.1126/scisignal.2004020 PMC4768865 23982205

[25] Gómez AM Henao CD Rebolledo M Jaramillo PP Muñoz VO Niño GL. Determination of time in range associated with hbA1c ≤7% in a prospective cohort of patients with type 1 diabetes using CGM for three months. J Diabetes Sci Technol. (2022) 18(2):345–50. doi: 10.1177/19322968221108424 PMC10973842 35791440

[26] Hu Y Han Y Liu Y Cui Y Ni Z Wei L. A nomogram model for predicting 5-year risk of prediabetes in Chinese adults. Sci Rep. (2023) 13:22523. doi: 10.1038/s41598-023-50122-3 38110661 PMC10728122

[27] Cai XT Ji LW Liu SS Wang MR Heizhati M Li NF. Derivation and validation of a prediction model for predicting the 5-year incidence of type 2 diabetes in non-obese adults: A population-based cohort study. Diabetes Metab syndrome obesity: Targets Ther. (2021) 14:2087–101. doi: 10.2147/dmso.S304994 PMC8123981 34007195

[28] Cai X Zhu Q Wu T Zhu B Aierken X Ahmat A. Development and validation of a novel model for predicting the 5-year risk of type 2 diabetes in patients with hypertension: A retrospective cohort study. BioMed Res Int. (2020) 2020:9108216. doi: 10.1155/2020/9108216 33029529 PMC7537695

[29] Zimmet P Alberti KG Magliano DJ Bennett PH. Diabetes mellitus statistics on prevalence and mortality: facts and fallacies. Nat Rev Endocrinol. (2016) 12:616–22. doi: 10.1038/nrendo.2016.105 27388988

[30] Peng LN Lin MH Lai HY Hwang SJ Chen LK Chiou ST. Risk factors of new onset diabetes mellitus among elderly Chinese in rural Taiwan. Age Ageing. (2010) 39:125–8. doi: 10.1093/ageing/afp193 19897541

[31] Kautzky-Willer A Harreiter J Pacini G. Sex and gender differences in risk, pathophysiology and complications of type 2 diabetes mellitus. Endocrine Rev. (2016) 37:278–316. doi: 10.1210/er.2015-1137 27159875 PMC4890267

[32] Defronzo RA. Banting Lecture. From the triumvirate to the ominous octet: a new paradigm for the treatment of type 2 diabetes mellitus. Diabetes. (2009) 58:773–95. doi: 10.2337/db09-9028 PMC2661582 19336687

[33] Han X Gao Y Qi W Ding S Zhang Y Xu Q. Influencing factors of coexistence PreDM and PreHTN in occupational population of state grid corporation of Chinese. Arch Environ Occup Health. (2020) 75:365–70. doi: 10.1080/19338244.2019.1703623 31847721

[34] Wu L Wu X Hu H Wan Q. Association between triglyceride-to-high-density lipoprotein cholesterol ratio and prediabetes: a cross-sectional study in Chinese non-obese people with a normal range of low-density lipoprotein cholesterol. J Trans Med. (2022) 20:484. doi: 10.1186/s12967-022-03684-1 PMC9588227 36273126

[35] Liu J Zhang Y Shi D He C Xia G. Vitamin D alleviates type 2 diabetes mellitus by mitigating oxidative stress-induced pancreatic β-cell impairment. Exp Clin Endocrinol diabetes: Off journal German Soc Endocrinol [and] German Diabetes Assoc. (2023) 131:656–66. doi: 10.1055/a-2191-9969 PMC10700019 37935388

[36] Anstee QM Targher G Day CP. Progression of NAFLD to diabetes mellitus, cardiovascular disease or cirrhosis. Nat Rev Gastroenterol Hepatol. (2013) 10:330–44. doi: 10.1038/nrgastro.2013.41 23507799

[37] Vitale M Xu C Lou W Horodezny S Dorado L Sidani S. Impact of diabetes education teams in primary care on processes of care indicators. Primary Care Diabetes. (2020) 14:111–8. doi: 10.1016/j.pcd.2019.06.004 31296470

[38] Chang X Wang Y Fu S Tang X Liu J Zhao N. The detection of thyroid nodules in prediabetes population and analysis of related factors. Risk Manage healthcare Policy. (2021) 14:4875–82. doi: 10.2147/rmhp.S337526 PMC8665774 34908885

[39] Buysschaert M Medina JL Bergman M Shah A Lonier J. Prediabetes and associated disorders. Endocrine. (2015) 48:371–93. doi: 10.1007/s12020-014-0436-2 25294012

[40] Zhang L Zhang Y Shen S Wang X Dong L Li Q. Safety and effectiveness of metformin plus lifestyle intervention compared with lifestyle intervention alone in preventing progression to diabetes in a Chinese population with impaired glucose regulation: a multicenter, open-label, randomized controlled trial. Lancet Diabetes Endocrinol. (2023) 11:567–77. doi: 10.1016/s2213-8587(23)00132-8 37414069

